# A novel anti-CD22 scFv–apoptin fusion protein induces apoptosis in malignant B-cells

**DOI:** 10.1186/s13568-017-0410-5

**Published:** 2017-06-02

**Authors:** Solmaz Agha Amiri, Soraya Shahhosseini, Najmeh Zarei, Dorsa Khorasanizadeh, Elahe Aminollahi, Faegheh Rezaie, Mehryar Zargari, Mohammad Azizi, Vahid Khalaj

**Affiliations:** 1 Department of Pharmaceutical Biotechnology, School of Pharmacy, Shahid Beheshti University of Medical Sciences, Tehran, Iran; 2 Department of Pharmaceutical Chemistry, School of Pharmacy, Shahid Beheshti University of Medical Sciences, Tehran, Iran; 3 Department of Medical Biotechnology, Biotechnology Research Center, Pasteur Institute of Iran, 13164 Tehran, Iran; 4 Department of Clinical Biochemistry, Molecular and Cell Biology Center, School of Medicine, Mazandaran University of Medical Sciences, Sari, Iran

**Keywords:** CD22, Single chain variable fragment, Apoptin, Fusion protein, Immunotherapy, Apoptosis

## Abstract

CD22 marker is a highly internalizing antigen which is located on the surface of B-cells and is being used as a promising target for treatment of B cell malignancies. Monoclonal antibodies targeting CD22 have been introduced and some are currently under investigation in clinical trials. Building on the success of antibody drug conjugates, we developed a fusion protein consisting of a novel anti-CD22 scFv and apoptin and tested binding and therapeutic effects in lymphoma cells. The recombinant protein was expressed in *E. coli* and successfully purified and refolded. In vitro binding analysis by immunofluorescence and flow cytometry demonstrated that the recombinant protein specifically binds to CD22 positive Raji cells but not to CD22 negative Jurkat cells. The cytotoxic properties of scFv–apoptin were assessed by an MTT assay and Annexin V/PI flow cytometry analysis and showed that the recombinant protein induced apoptosis preferentially in Raji cells with no detectable effects in Jurkat cells. Our findings indicated that the recombinant anti-CD22 scFv–apoptin fusion protein could successfully cross the cell membrane and induce apoptosis with high specificity, make it as a promising molecule for immunotherapy of B-cell malignancies.

## Introduction

Hematological malignancies are heterogeneous types of cancer and include a significant population of cancer patients. Among these, B-cell malignancies are more common with an incidence rate of 21 per 100,000 (Keppler-Hafkemeyer et al. 2000; Sant et al. 2010; Weber et al. 2015). Treatment outcomes in B-cell neoplasms have been greatly improved due to the availability of effective prognostic factors and the optimal use of conventional chemotherapeutic agents in combination with tumor specific monoclonal antibodies (mAbs) and stem cell transplantation (Hennessy et al. 2004; Pui and Evans 2006). However, some patients suffer from treatment refractory responses or even adverse effects related to the treatment (Coiffier et al. 2002; Raut and Chakrabarti 2014). Therefore, there is a need to develop novel and effective therapies with increased antitumor efficacy. The targeted delivery of cytotoxic agents to the tumor cells using tumor-specific antibodies is an effective treatment strategy which has been clinically validated using multiple approaches. Full-length antibodies or antibody fragments targeting a variety of tumor antigens have been combined with radionuclides, cytotoxic proteins, bacterial toxins, or chemotherapeutic drugs for cancer treatment (Becker and Benhar 2012; Kreitman and Pastan 2011; Noteborn 2009).

Single-chain variable fragments (scFvs) are the smallest functional fragments of immunoglobulins that maintain the antigen-binding specificity of the parental molecule. These molecules are comprised of variable heavy (V_H_) and variable light (V_L_) chains of an antibody that are joined by a flexible polypeptide linker (Monnier et al. 2013; Nelson 2010).

In comparison with intact antibodies, scFvs, with a molecular weight of ~30 kDa, have many advantages for therapeutic purposes. For instance, they demonstrate an enhanced tissue penetration and distribution as well as rapid clearance from circulation (Malpiedi et al. 2013; Weisser and Hall 2009). These antibody fragments can be produced more economically and easily engineered as fusions with cytotoxic proteins, drugs, or radionuclides (Ahmad et al. 2012; Malpiedi et al. 2013).

CD22 is a ~140-kDa type 1 membrane glycoprotein that belongs to sialoadhesin protein family (Nitschke 2005; Tedder et al. 2005), and is a B-cell-specific surface antigen that modulates function, survival, and apoptosis of B-cell (Nitschke 2005; Sullivan-Chang et al. 2013). Several characteristics make CD22 an attractive target for immunotherapy of B-cell malignancies. First, it is expressed on the surface of mature B-cells with low level of expression in precursor B-cells and no expression in mature plasma cells. Second, CD22 is rapidly internalized following the ligand attachment, making it effective for intracellular delivery of diagnostic and therapeutic cargo (Alderson et al. 2009; Tedder et al. 2005; Vallera et al. 2005b). Third, CD22 expression is present in 60–80% of B-cell lymphomas or leukemias (Alderson et al. 2009; Cesano and Gayko 2003), including hairy cell leukemia (HCL), chronic lymphoblastic leukemia (CLL), non-Hodgkin’s lymphoma (NHL), and acute lymphoblastic leukemia (ALL) (Kreitman and Pastan 2011). Both conjugated and unconjugated anti-CD22 antibodies have been developed for treatment of B-cell neoplasms, and some of them are currently in clinical trials (Kreitman and Pastan 2011; Teo et al. 2016).

Apoptin is a small (14 kDa) apoptosis-inducing protein encoded by VP3 gene of chicken anemia virus (CAV) which can induce apoptosis in various human tumors or transformed cells, but not in primary non-transformed cells (Danen-Van Oorschot et al. 1997; Los et al. 2009; Maddika et al. 2006). However, the exact mechanism(s) of apoptin-induced cell death and its ability to distinguish tumor cells from normal cells remains unclear (Bullenkamp and Tavassoli 2014; Los et al. 2009). The cancer-selective toxicity of apoptin is largely related to differences in the subcellular localization of the protein. Apoptin is predominantly accumulated in the nucleus of transformed cells, whereas in normal cells it is found mainly in the cytoplasm (Heilman et al. 2006; Kuusisto et al. 2008; Noteborn 2004; Peñaloza et al. 2014). Apoptin can be phosphorylated in several phosphorylation sites by certain kinases, which are upregulated in tumor cells. The phosphorylation process is important for the nuclear accumulation and apoptotic function of apoptin molecule (Bullenkamp et al. 2015; Danen-van Oorschot et al. 2003; Kuusisto et al. 2008; Peñaloza et al. 2014).

In the present study, we generated a recombinant anti-CD22 scFv–apoptin fusion protein as a bifunctional molecule: the anti-CD22 scFv moiety targets the CD22 antigen on malignant B-cells surface and apoptin specifically kills the tumor cells. We utilized *Escherichia coli* BL21 (DE3) as the preferred system for expression of the antibody fragment because of the rapid growth rate, inexpensive substrates, well-known genetics, and easy manipulation (Ahmad et al. 2012; Weisser and Hall 2009). Functional assays were performed to assess the targeting properties and specificity of the fusion protein in CD-22 positive and negative cells. Furthermore, the toxic properties of the fusion protein were examined to identify the potency of this novel tumor-targeting bioconjugate.

## Materials and methods

### Bacterial strains, cell lines and plasmids

*E. coli* strains Top 10F′ and BL21 (DE3) were used as hosts for plasmid preparation and recombinant protein expression, respectively. These *E. coli* strains and the protein expression vector pET-28a (+) were purchased from invitrogen (Carlsbad, CA, USA). pGEM–T Easy (Promega, Madison, WI, USA) was used as the intermediate vector throughout the cloning steps. *E. coli* strains were grown in Luria–Bertani (LB) medium [1% (w/v) tryptone, 0.5% (w/v) yeast extract, and 1% (w/v) NaCl, pH 7.0]. The growth medium was supplemented with the antibiotics ampicillin (100 µg/mL; for *E. coli* Top 10F′) and kanamycin [50 µg/mL; *E. coli* BL21 (DE3)] when required. Restriction endonucleases were obtained from Fermentas (Waltham, USA). T4 DNA ligase was purchased from Roche (Penzberg, Germany). Primers were synthesized by SinaClon BioScience (Tehran, Iran). All chemicals and reagents used were provided from standard commercial sources.

Mycoplasma free hematopoietic Raji (CD22^+^) and Jurkat cell (CD22^−^) lines were obtained from National Cell Bank of Iran (NCBI), Pasteur institute of Iran. The cell lines were cultured in RPMI 1640 complete medium supplemented with 10% (v/v) fetal bovine serum (FBS) and 1% penicillin–streptomycin (100 U/mL penicillin and 100 µg/mL streptomycin), at 37 °C under 5% CO2 in a humidified incubator.

### Construction of anti-CD22 scFv–apoptin cassette

The scFv gene was PCR-amplified from an intermediate plasmid pGH–scFv, containing the anti-CD22 scFv sequence (Zarei et al. 2014). The specific primers scFv-forward 5′-CCATGGAAAAGAGAGGCTG-3′; containing the *Nco*I recognition sequence (underlined) and scFv-reverse 5′-GGCGGCCGCTCTCTTGATCTCCAAC-3′; containing the *Not*I recognition sequence (underlined) were used in the PCR reaction. PCR was performed using the expand high fidelity PCR system (Roche, Penzberg, Germany) in a 50 µL mixture volume according to the manufacturer’s protocol. The resulting PCR product was gel-purified using QIAquick Gel Extraction Kit (Qiagen, Hilden, Germany). The purified fragment was then cloned into the pGEM-T easy vector to generate pGEM–scFv construct.

The codon optimized sequence encoding apoptin protein was synthesized based on its amino acid sequence (GenBank: AEB91668.1) (GeneRay Biotech,Shanghai, China). The sequence was modified to include a 5′ (Gly_4_Ser)_3_ linker sequence to the fused apoptin protein downstream of the *anti*-*CD22 scFv* sequence, and a C-terminal 6XHis-tag was added to facilitate the later purification and immunodetection of the fusion protein. The synthetic fragment flanked by *Not*I enzyme site at the 5′ terminal and *Hin*dIII enzyme site at the 3′ terminal and cloned into the pGH vector.

To construct pGEM–scFv–apoptin, pGEM–scFv and pGH–apoptin were digested simultaneously with *Not*I/*Hin*dIII, and after gel purification, *apoptin* fragment was cloned into *Not*I/*Hin*dIII restriction sites of pGEM–scFv Vector.

For preparation of the expression construct, the whole gene fragment encoding N-terminal scFv and C-terminal apoptin (GeneBank accession number: KY884983) was digested with *Nco*I/*Hin*dIII from pGEM–scFv–apoptin plasmid and cloned into the *Nco*I/*Hin*dIII site of pET-28a (+). The final construct was named as pET–scFv–apoptin and transformed into competent Top 10F′ cells. The positive transformants were selected on LB agar with 50 µg/mL kanamycin. The recombinant plasmid was verified with colony PCR, restriction mapping and DNA sequencing. All DNA manipulations were performed using the standard techniques.

### Small scale expression of recombinant scFv–apoptin

The *E. coli* BL21 (DE3) competent cells were transformed with the recombinant plasmid. A single colony of the transformed strain was selected and protein expression was induced by isopropyl β-D-1-thiogalactopyranoside (IPTG) (Sigma–Aldrich, St. Louis, USA) at a final concentration of 1 mM. Following the induction step, the bacterial biomass was collected by centrifugation, resuspended in TE buffer (50 mM Tris–HCl, 1 mM EDTA, 100 mM NaCl, pH 8.0) and disrupted by sonication. Then, the suspension of disrupted cells was centrifuged at 10,000*g* for 20 min at 4 °C to separate soluble and insoluble fractions. Finally the fractions were analyzed on a 12% sodium dodecyl sulfate–polyacrylamide gel electrophoresis (SDS-PAGE) gel. In addition, *E. coli* BL21 (DE3) was transformed with only pET-28a (+) vector to use in parallel as a negative control. Protein expression levels were quantified based on SDS-PAGE images using Quantity One 4.62 software (Bio-Rad laboratories, Hercules, CA, USA).

### Western blot analysis

For western blotting, equivalent amounts of samples were resolved on a 12% SDS-PAGE and the separated bands were transferred to a polyvinylidene difluoride (PVDF) membrane. The PVDF membrane was blocked with 5% skimmed milk powder in phosphate buffer saline (PBS), then immunoblotted with a HRP labeled anti-His-tag antibody (Roche, Penzberg, Germany). The positive bands were detected by using an enhanced chemiluminescence detection system (Amersham Life Science, Buckinghamshire, UK).

### Large scale protein production

Large scale recombinant protein production was performed by inoculating a single colony into 30 mL LB broth containing 50 µg/mL kanamycin at 37 °C overnight with shaking. The overnight culture was inoculated in 1 L LB medium supplemented with the antibiotic at 37 °C with shaking until an OD_600_ of approximately 0.6 was reached. Protein expression was induced by addition of IPTG at the final concentration of 1 mM. The cells were grown for an additional overnight at 37 °C before harvesting by centrifugation at 10,000 rpm for 30 min at 4 °C. Pellets were stored at −70 °C until required.

### Purification and refolding of recombinant scFv–apoptin

Purification of recombinant His-tagged scFv–apoptin from inclusion bodies was carried out by immobilized metal affinity chromatography (IMAC) using agarose bead technologies (ABT) resin (Madrid, Spain), based on the manufacturer’s instructions for denaturating condition. The scFv–apoptin contained fractions were pooled and subjected to refolding step via dialysis to remove urea and imidazole. The dialysis procedure was performed using a continuous gradient of urea from 8 to 0.5 in an exchange buffer (50 mM NaH_2_Po_4_, 300 mM NaCl, 250 mM imidazole, pH 8.0) and finally against phosphate buffer (pH 7.0, containing 1 mg/mL BSA) at 4 °C. The refolded protein solution was centrifuged to remove aggregates at 10,000*g* for 15 min at 4 °C and concentrated by ultrafiltration. The purity of the recombinant protein was analyzed by SDS-PAGE at each step of purification and refolding. Bradford assay was used to determine the protein concentration of each fraction using the bovine serum albumin (BSA) as the protein standard. The protein samples were stored at −70 °C until required.

### MTT assay

The cytotoxicity of the recombinant scFv–apoptin was determined by MTT (3-(4,5-dimethylthiazol-2-yl)-2,5-diphenyltetrazolium bromide) (Sigma–Aldrich, St. Louis, USA) assay. Briefly, Raji and Jurkat cells were seeded at a density of 1 × 10^4^ cells/well into 96-well flat-bottomed plates (at 50 µL/well). After 4 h pre-incubation, indicated concentrations of the recombinant protein were added to the triplicate wells containing cells and incubated for 24, 48 and 72 h. In addition, purified scFv in PBS (Zarei et al. 2014) was used as a control for scFv–apoptin in the same way. The cells were also treated with cisplatin at concentration of 10 µg/mL and fresh medium as positive and negative control, respectively. Then, 20 µL of 5 mg/mL MTT solution in PBS was added into each well and incubated for another 4 h for purple crystal formation. After incubation, the precipitated crystals were dissolved using 150 µL DMSO for 20 min.

Finally, the OD was measured at 545 nm as detection wavelength and 630 nm as reference wavelength with a multiple scanning spectrophotometer (ELISA reader, Organon Tekninka, Oss, Netherlands). Cell viability percentage was calculated using the following formula:

$${\text{Cell viability }}\left( \% \right) = {{\left( {{\text{OD of treated group}}-{\text{OD Blank}}} \right)} \mathord{\left/ {\vphantom {{\left( {{\text{OD of treated group}}-{\text{OD Blank}}} \right)} {\left( {{\text{OD of control group}} - {\text{OD Blank}}} \right) \times 100}}} \right. \kern-0pt} {\left( {{\text{OD of control group}} - {\text{OD Blank}}} \right) \times 100}}$$

The inhibitory concentration 50% (IC_50_) value of scFv–apoptin was defined as the concentration of recombinant protein that induce 50% reduction in cellular viability and it was calculated by means of GraphPad Prism software (Version 6.07).

### Flow cytometry binding analysis

To assess the specific binding of the recombinant scFv–apoptin to the CD22 surface antigen, the CD22^+^ Raji cells were exposed to the fusion protein and the binding was evaluated by FACS analysis as described before (Zarei et al. 2014). CD22^**−**^ Jurkat cells were also used as negative control. In brief, after blocking and washing steps, both Raji and Jurkat cells were incubated with 5 µg of the recombinant protein for 45 min in 4 °C. At the next step, 1 µg of mouse anti-His-tag monoclonal antibody was added and the cell suspension was kept for 30 min at 4 °C. Then, the cells were incubated with 1:32 diluted Fluorescein isothiocyanate (FITC)-labeled goat anti-mouse IgG (Razi, Tehran, Iran) for 30 min. In parallel, each cell line was also incubated with 500 ng of RFB4 (Santacruz Biotechnology, CA, USA) or isotype control IgG1 (according to manufacturer’s recommendation), for 45 min at 4 °C and passed through washing steps. Subsequently, the cells were incubated with a 1:32 dilution of the FITC anti-mouse IgG at 4 °C in the dark and washed with FACS buffer to remove unconjugated antibodies and finally resuspended in fixing buffer (PBS, 1% paraformaldehyde). In addition, untreated Raji and Jurkat cells were incubated with anti-6XHis tag and FITC anti-mouse IgG, to evaluate the background fluorescence following the same condition described above. Flow cytometry was carried out using a CyFlow^®^ SL machine (Partec, Munster, Germany) and mean fluorescence intensity (MFI) values were determined using Flomax^®^ software (Partec, Munster, Germany). To assess competitive binding of RFB4 (Santacruz, CA, USA) in the presence of the scFv–apoptin, Raji cells were first incubated with 10 µg of the recombinant protein for 1 h at 4 °C. After two times washing with FACS buffer, 500 ng of the mAb RFB4 was added and incubated for 1 h at 4 °C, washed again with FACS buffer and further incubated with FITC labeled anti mouse IgG (1:32). Evaluation of RFB4 binding inhibition to the Raji cells in the presence of competing scFv–apoptin was performed using calculation of maximum MFI percentage of RFB4 in the absence of competing scFv–apoptin.

### Immunofluorescence scFv–apoptin binding assay

Further binding evaluation and internalization assessment of anti-CD22–scFv–apoptin were performed in Raji and Jurkat cells using immunofluorescence staining as described before (Zarei et al. 2014). Five microgram of the recombinant protein was used in this assay. Non-treated Raji and Jurkat cells used as negative controls. As a positive control, Raji and Jurkat cells were treated with 500 ng RFB4 mAb and the binding assay was investigated similarly. Finally, the emitted fluorescence was visualized using a fluorescence microscope (Jenus, China).

### Flow cytometric apoptosis assay

To assess the specific apoptotic activity of the recombinant scFv–apoptin, a flow cytometry analysis was performed. Raji and Jurkat cells were seeded at 2 × 10^5^ cells/well in 6 well plates, then the cells were treated with 30 µg/mL of the recombinant protein or appropriate volume of PBS as control. After 24, 48 and 72 h of incubation, 10^6^ cells were harvested by centrifugation at 200*g* for 5 min and washed once with cold PBS. Subsequently cell pellets were resuspended in 100 µL of annexin-V-Fluos labeling solution that prepared according to Annexin-V-FLUOS staining kit instruction (Roche, Penzberg, Germany). Cells were then incubated at 15–25 °C for 10–15 min in the dark and then assayed by flow cytometry. Live cells populations (Annexin V^−^/PI^−^), early apoptotic cells (Annexin V^+^/PI^−^) and late apoptotic or dead cells (Annexin V^+^/PI^+^) percentage was measured using a CyFlow^®^ SL machine (Partec, Munster, Germany). The percentage of specific apoptosis was calculated as follows:

$$\% {\text{Specific apoptosis}} = {{ \left[ {\left( {\% {\text{induced apoptosis in the assay well}}} \right) - \left( {\% {\text{spontaneous apoptosis in the control well}}} \right) \times 100} \right]} \mathord{\left/ {\vphantom {{ \left[ {\left( {\% {\text{induced apoptosis in the assay well}}} \right) - \left( {\% {\text{spontaneous apoptosis in the control well}}} \right) \times 100} \right]} { 100 - \left( {\text{spontaneous apoptosis in the control well}} \right)}}} \right. \kern-0pt} { 100 - \left( {\text{spontaneous apoptosis in the control well}} \right)}}$$

(Fulda et al. 1997).

Each sample was run in triplicates.

### Statistical analysis

All experiments were repeated at least three times, and the results are expressed as the mean ± standard deviation (SD). The statistical analysis of differences between groups in MTT assay was performed by one-way analysis of variance (ANOVA) followed by a post hoc Tukey test for intergroup comparisons. For apoptosis studies, differences between groups were analyzed by unpaired t test. *P* value <0.05 was considered as statistically significant in all the experiments.

## Results

### Expression analysis of the recombinant scFv–apoptin

**Fig. 1:**
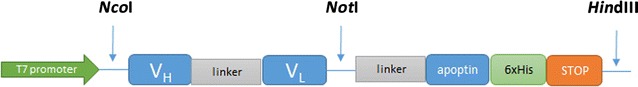
A schematic diagram of anti-CD22 scFv–apoptin construct. The orientation of fragments used in the construct has been shown

**Fig. 2:**
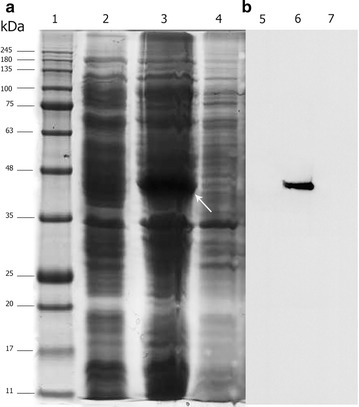
The expression analysis of anti-CD22 scFv–apoptin in the soluble and insoluble fractions of lysed bacterial cells. **a** SDS-PAGE analysis: protein molecular weight standards (*lane 1*), total protein from *E. coli* BL2 (DE3) containing pET-28 a (+) vector (negative control) grown under identical condition (*lane 2*), insoluble fraction (*lane 3*), and supernatant of cell lysate (*lane 4*) of induced *E. coli* BL2 (DE3) containing pET-anti-CD22 scFv–apoptin. A major band of ~44 kDa in insoluble fraction indicates anti-CD22 scFv–apoptin (*white arrow*). **b** Western blot analysis: the samples were loaded as in **a**

The final expression cassette, pET–scFv–apoptin, was sequenced and the results confirmed that the *scFv* gene fragment was in V_H_ to V_L_ orientation and the (Gly_4_Ser)_3_ linker coding sequence was located between two chains. A second linker also was present to fuse *apoptin* gene to the 3′ end of the *scFv* gene fragment. A His-tag sequence was also inserted at the 3′ region of the *apoptin* gene before the stop codon (data not shown and Fig. 1). The construct was transformed in *E. coli* as described and the expression analysis of the recombinant anti-CD22–scFv–apoptin was carried out after 6 h of IPTG induction. As it is shown in Fig. 2a, the scFv–apoptin fusion protein migrated approximately at 44 kDa which corresponds to its predicted molecular weight. No similar band was detected in soluble fraction. The presence of an immunoreactive band of ~44 kDa in western blot analysis of insoluble fraction confirmed the expressed protein (Fig. 2b). These findings revealed that the recombinant scFv–apoptin was successfully expressed mainly in insoluble form in *E. coli* BL21 (DE3).

### Large scale protein production, purification and refolding

For large scale preparation of the recombinant protein, 1 L of bacterial culture was induced under the optimum condition. The final wet weight of the collected biomass was approximately 4.4 g/L. The recombinant protein was expressed as insoluble inclusion bodies, making up 15% of total protein based on densitometry analysis. The solubilized inclusion bodies were purified under the denaturating condition and all collected fractions were analyzed by SDS-PAGE and western blot which confirmed the authenticity of the refolded protein. The final purity, recovery yield and the amount of concentrated protein was 91%, 19% and 2.7 mg, respectively.

### MTT assay

**Fig. 3:**
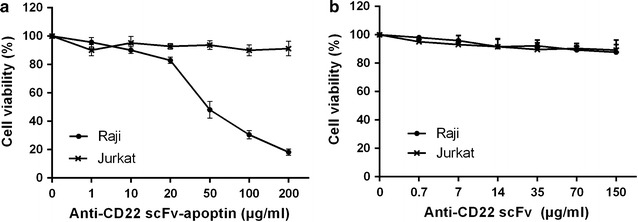
In vitro dose dependent cytotoxicity curves of anti-CD22 scFv–apoptin and anti-CD22 scFv on the CD22 positive Raji cells and CD22 negative Jurkat cells. The cells were incubated with different concentrations of anti-CD22 scFv–apoptin (**a**) and anti-CD22 scFv (**b**) for 72 h, respectively. The cell viability was measured using standard MTT assay. Data are represented as mean ± standard deviation, from 3 experiments performed in triplicates

**Fig. 4:**
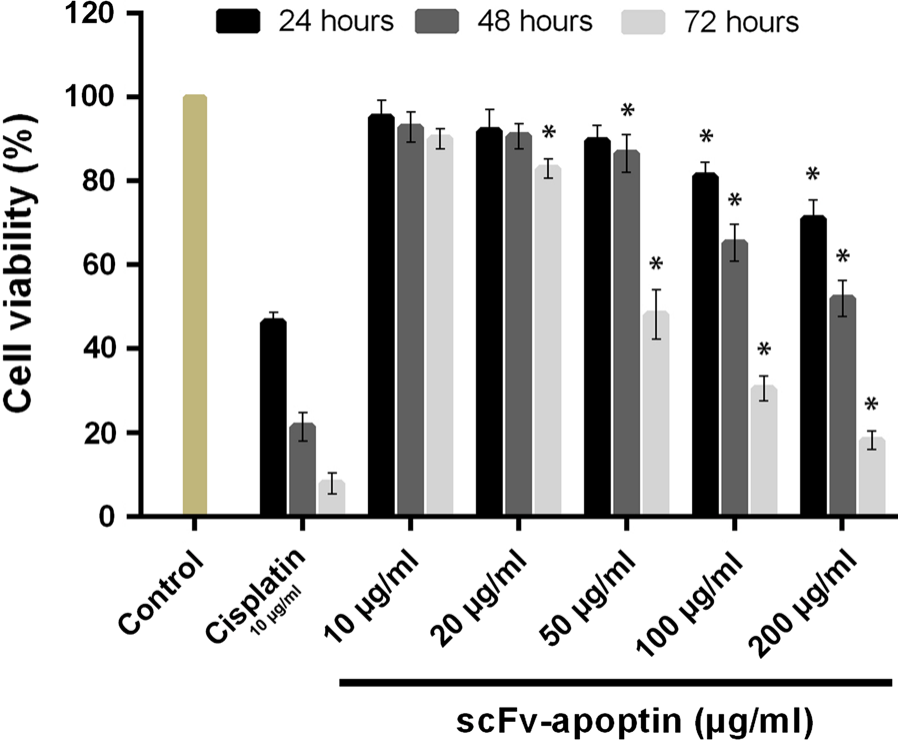
Cytotoxicity of anti-CD22 scFv–apoptin against Raji cells at 10, 20, 50, 100 and 200 µg/mL of the protein concentrations. Incubation time was 24, 48 and 72 h. Data are represented as mean ± standard deviation from 3 experiments performed in triplicates (*p < *0.05*)

In vitro cytotoxic effect of the recombinant protein on CD22 positive Raji cells and CD22 negative Jurkat cells was evaluated by MTT assay. The cells were treated with increasing concentrations of the recombinant scFv–apoptin (1, 10, 20, 50, 100 and 200 µg/mL) or recombinant scFv (0.7, 7, 14, 35, 70 and 150 µg/mL), and cell viability was determined after 24, 48 and 72 h of exposure. scFv–apoptin reduced cell viability of Raji cells significantly (*p* < 0.05) in a dose dependent manner (IC_50_: 43.67 µg/mL, exposure time 72 h), while no significant inhibition was observed in Jurkat cells (Fig. 3a). The scFv protein did not cause notable cytotoxic effect in either cell line even at high concentration after 72 h, indicating a lack of toxicity (Fig. 3b). Figure 4 shows the percentage of viable Raji cells incubated with various concentrations of the recombinant protein for 24, 48 and 72 h. At lower concentrations (up to 10 µg/mL), there was no cytotoxic effect on the Raji cells over 72 h. However, at higher concentrations (20, 50, 100 and 200 µg/mL), a significant dose and time dependent decrease in cell viability was seen (*p* < 0.05). Also, after 24 h incubation, a slight decrease was seen in cell viability and only concentrations of 100 and 200 µg/mL were significant (*p* < 0.05). Conversely, a significant reduction in cell viability was observed at concentrations of 50–200 µg/mL up to 48 h (*p* < 0.05). At exposure times over 72 h, the concentrations of 100 and 200 µg/mL strongly affected cell viability while concentrations of 20 and 50 µg/mL of the recombinant protein also exhibited cytotoxicity in Raji cells. As seen in Fig. 4, cisplatin treatment of Raji cells reduced viability by >50% at 24 h of induction. Collectively, these findings demonstrated that scFv–apoptin can selectively reduce the cell viability in CD22^+^ cells in a time and dose-dependent manner.

### Flow cytometry analysis of cell binding

**Fig. 5:**
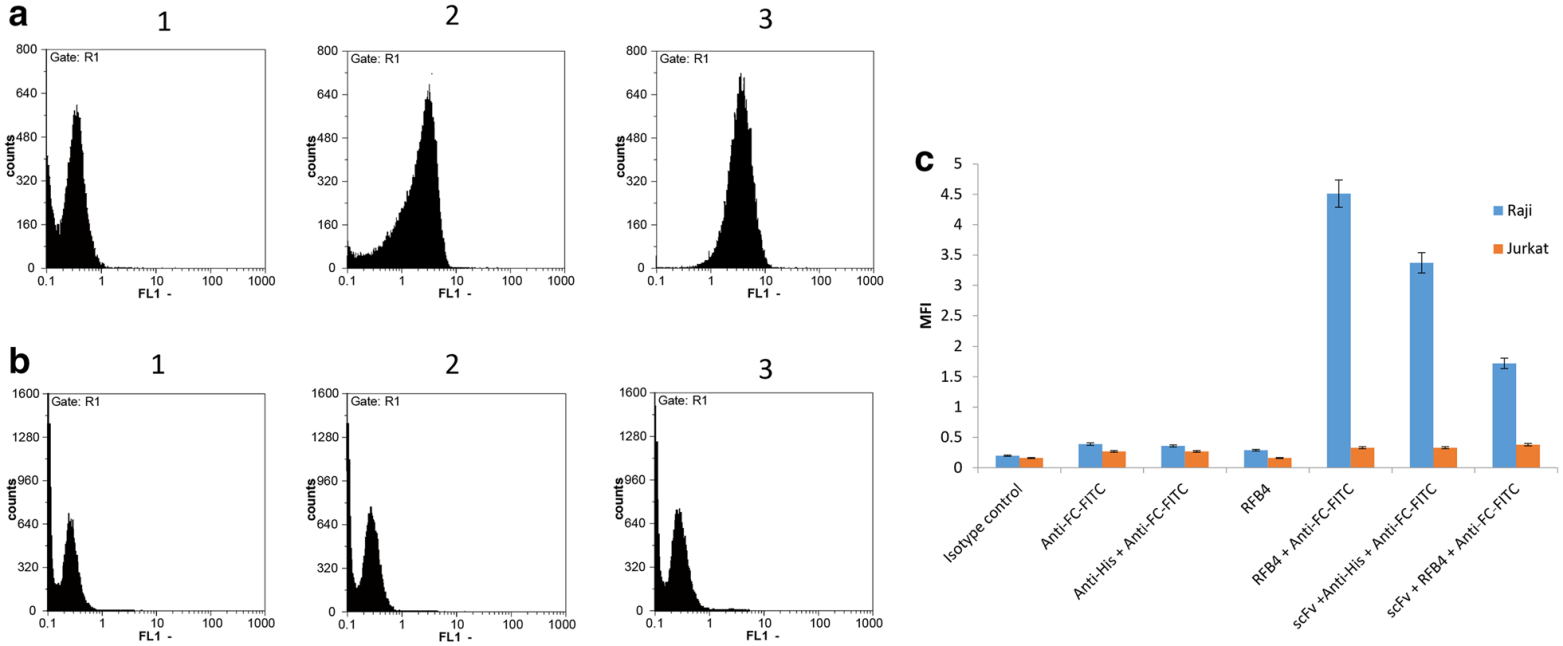
The flow cytometry analysis of scFv–apoptin binding to Raji or Jurkat cells. The binding of isotype control (*1*), scFv–apoptin (*2*), and RFB4 (*3*), to the cell surface of Raji (**a**) and Jurkat (**b**) cells is shown. The recombinant scFv–apoptin specifically binds to the CD22 positive Raji cells as presented by shift in fluorescence value in Raji cells in comparison with the lower value of the fluorescence intensity measured from the Jurkat cells. **c** Plot of the MFI value of Raji and Jurkat cells incubated with presented antibodies

Flow cytometry analysis was performed to examine whether the purified scFv–apoptin was able to recognize the CD22 antigen on the cell surface of the CD22^+^ cells. As presented in Fig. 5a, b, anti-CD22 scFv–apoptin was able to bind specifically to Raji cells as shown by a shift in fluorescence intensity value when compared to Jurkat cells. As a positive control, a commercial anti-CD22 antibody, RFB4, was used in parallel. As expected, Raji cells showed higher binding of RFB4 (MFI value of 4.51) compared with Jurkat cells (MFI value of 0.33) (Fig. 5c). Competition studies showed that pre-incubation of Raji cells with the recombinant protein caused the MFI value of RFB4 to decrease by 60% (1.72 blocked vs. 4.51 unblocked), suggesting that the fusion protein recognizes the same epitope of CD22 receptor as the RFB4 antibody.

### Immunofluorescence analysis of the targeting specificity of scFv–apoptin

**Fig. 6:**
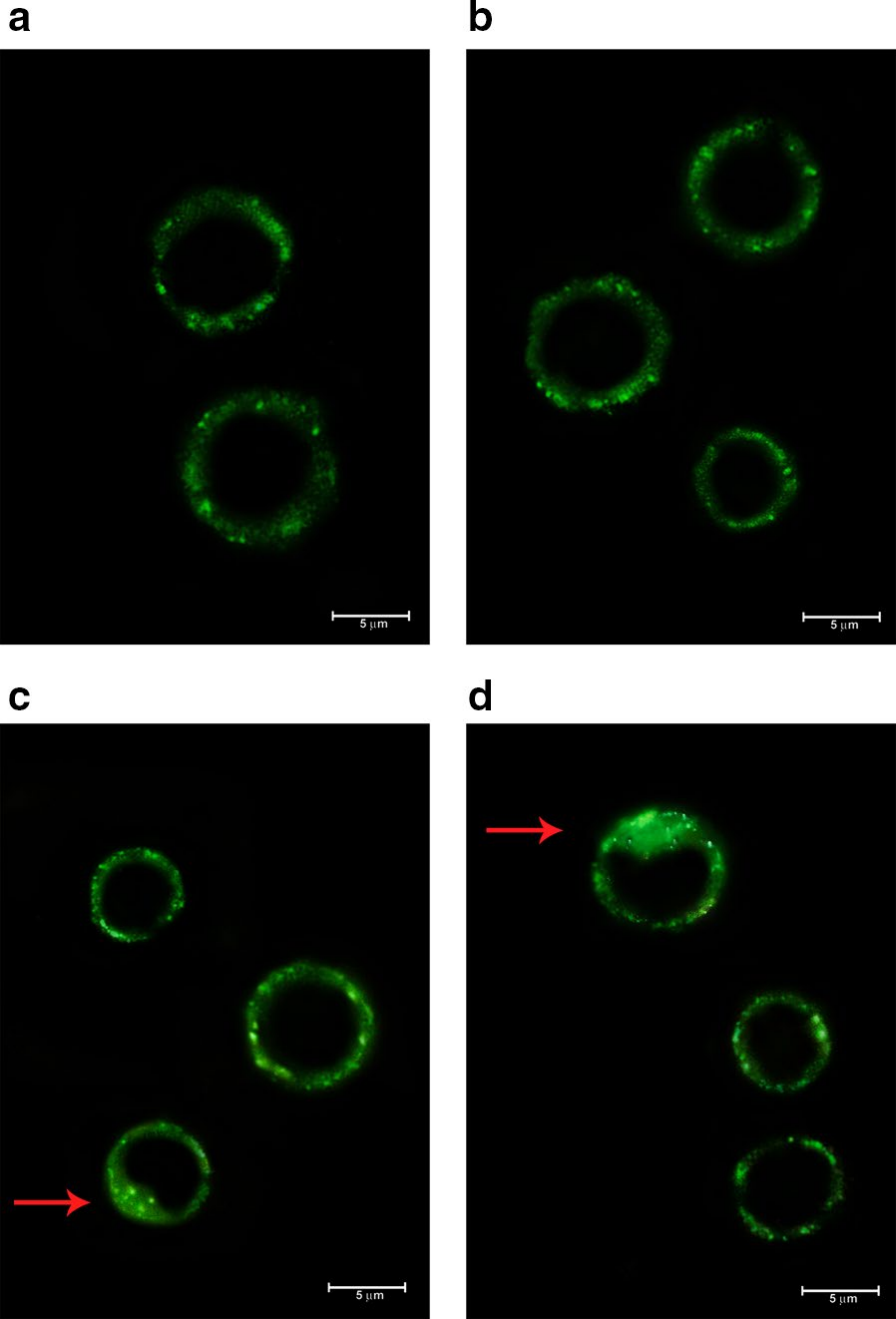
Immunofluorescence staining of Raji cells for confirmation of binding activity and internalization of scFv–apoptin. Raji cells were incubated with scFv–apoptin at 4 °C (5 µg/mL) (**a**), RFB4 at 4 °C (500 ng/mL) (**b**), scFv–apoptin at 37 °C (**c**), and RFB4 at 37 °C (**d**). After 2 h of incubation, cells were washed, fixed, permeabilized and treated with anti-6XHis-tag antibody and FITC anti-mouse IgG. Observation under the fluorescence microscope revealed cell surface binding and internalization (indicated by *arrows*) of the fusion protein

The specific binding and internalization of the recombinant scFv–apoptin into the target cells through the scFv, is essential for effective delivery of apoptin and its potential therapeutic function. scFv–apoptin binding and subsequent internalization in Raji cells were assessed by comparing scFv–apoptin cell association at 4 °C versus 37 °C. At 4 °C, robust membrane staining was observed (Fig. 6a) while internalization occurred at 37 °C (Fig. 6c), indicating that the purified scFv–apoptin could successfully bind to CD22 receptors on Raji cells and accumulate within the cells. No staining was observed in Jurkat cells under the same conditions (data not shown).

### Apoptosis induction by scFv–apoptin

**Fig. 7:**
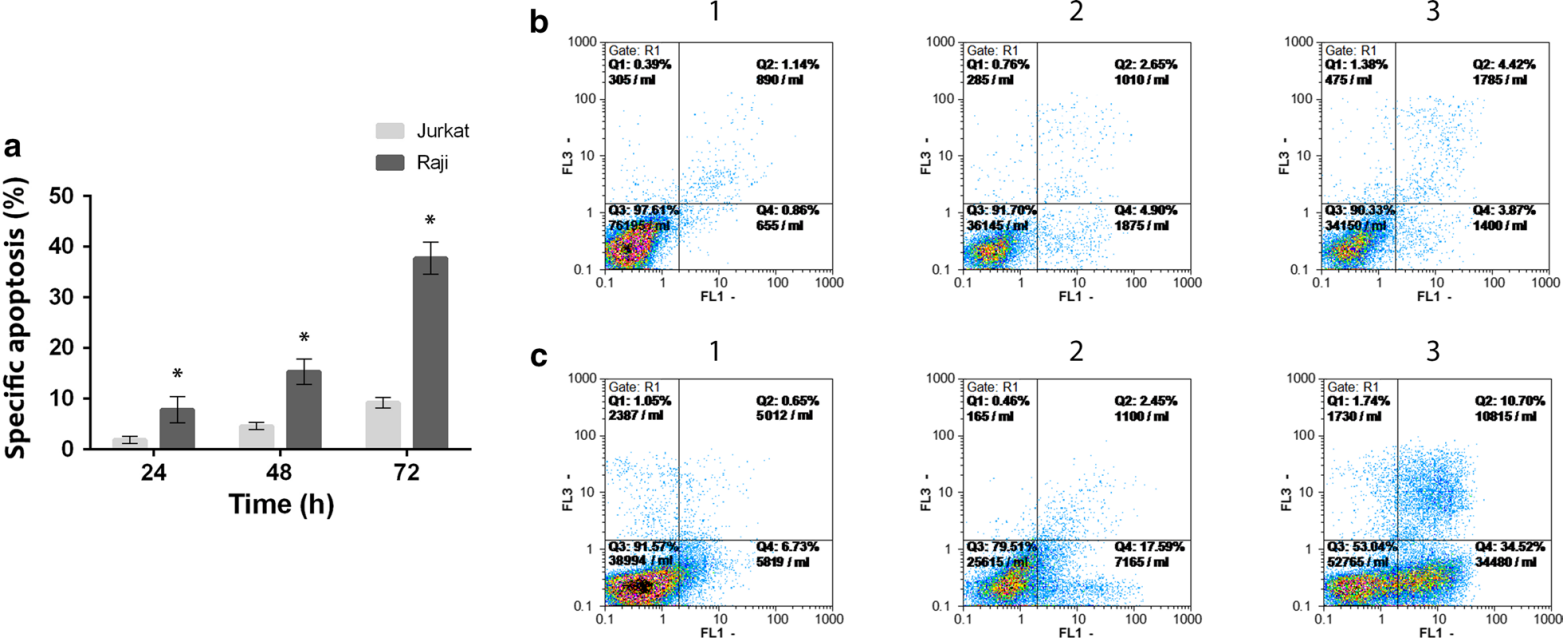
Apoptosis induced by scFv–apoptin in Raji and Jurkat cells assayed by Annexin-V/PI staining. **a** Percentage of specific apoptosis in Raji and Jurkat cells incubated with recombinant scFv–apoptin. 24, 48 and 72 h later, Annexin-V/PI staining was quantified by flow cytometric analysis. The rate of specific apoptosis in the Raji cells was significantly higher than that of Jurkat cells. Data represents mean ± standard deviation from 3 independently performed experiments (*p < *0.05*). Flow cytometric graphs of un-treated (**b**) and scFv–apoptin treated (**c**) Raji cells incubated for 24 (1), 48 (2) and 72 (3) h. early apoptotic cells are Annexin-V-positive, PI-negative (*lower right*), whereas late apoptotic cells are Annexin-V positive and PI-positive (*upper right*)

To identify if scFv–apoptin-induced cell death observed in the MTT assay occurs through the apoptosis process, the proportion of early and late apoptotic cells were examined by Annexin-V/PI flow cytometric analysis. The results showed that scFv–apoptin significantly induced a higher level of specific apoptosis in Raji cells compared to Jurkat cells in a time-dependent manner (*p* < 0.05) (Fig. 7a). As shown in Fig. 7a and b, the percentage of specific apoptosis in Raji cells was 7.8 ± 2.5% at 24 h and increased to 15.3 ± 2.5% at 48 h. The maximum frequency of apoptotic Raji cells was reached at 72 h (37.7 ± 3.1%) while slight early/late apoptosis induction (9.2 ± 1%) was seen in Jurkat cells. These findings demonstrated the ability of the scFv–apoptin protein to successfully induce apoptosis in Raji cells.

## Discussion

Antibody targeted therapy in both lymphoid and myeloid hematological malignancies has advanced significantly in recent years (Tu et al. 2011; Wayne et al. 2014). Five Out of 12 approved therapeutic monoclonal antibodies (mAbs) for cancer therapy are being used in the treatment of lymphoma or leukemia (Firer and Gellerman 2012). Naked therapeutic mAbs successfully bind to the specific surface antigens of tumor cells and kill them through induction of apoptosis, antibody dependent cytotoxicity (ADCC) and complement dependent cytotoxicity (CDC) (Potala et al. 2008), however, based on current evidences, using unconjugated mAbs alone is rarely potent in some types of hematological malignancies and they are usually administrated in combination with chemotherapeutic drugs. Several approaches have been used to overcome this problem, one of these is using highly specific mAbs to deliver cytotoxic substances to the cancer cells to enhance cell killing (Becker and Benhar 2012; FitzGerald et al. 2011). Choice of target antigen is critical in the development of these bifunctional molecules. The ideal antigen must be highly expressed on the surface of malignant cells compared with the normal cells, antigen should have minimum shedding to prevent antigen–antibody binding within blood circulation, and be effectively internalized after binding to the specific antibody (Diamantis and Banerji 2016; FitzGerald et al. 2011).

Throughout the last decade, CD22 has been served as an effective therapeutic target for the treatment of B-cell malignancies due to its restricted expression on B-cells and also its rapid internalization upon binding to its ligands or antibody (Teo et al. 2016; Tu et al. 2011). Several immunotherapeutic agents has been developed that selectively target CD22 antigen in either naked format (Carnahan et al. 2003; Furman et al. 2004; Leonard et al. 2004) or chimeric proteins fused with protein payloads like immunoenzymes (Krauss et al. 2005, 2009; Weber et al. 2015), immunotoxins with bacterial (Alderson et al. 2009; Kreitman and Pastan 2011; Vallera et al. 2005b) or plant (Messmann et al. 2000) toxins, or combined with non-protein therapeutic payloads such as radioactive isotopes (Vallera et al. 2005a) or chemotherapeutic drugs as antibody drug conjugates (ADCs) (DiJoseph et al. 2004, 2005). Clinical trials in patients with NHL are now ongoing with anti-CD22 antibody drug conjugates or unconjugated (DiJoseph et al. 2004, 2005; Leonard et al. 2004). These immunotherapeutic agents are being developed as humanized whole antibodies, while in another approach an antibody fragment have been used to develop moxetumomab pasudotox, an immunotoxin composed of a variable fragment (Fv) of the anti-CD22 antibody, RFB4, fused to the truncated pseudomonas exotoxin and is being evaluated in phase III in patients with hairy cell leukemia (Kreitman and Pastan 2011, 2015; Teo et al. 2016). Recently, a class of viral and cellular proteins encoded by anticancer genes has been revealed that cause cancer specific cell killing. Apoptin, a small protein derived from chicken anemia virus (CAV) was the first one discovered. This protein induces apoptosis specifically in tumor cells while sparing normal cells (Grimm and Noteborn 2010; Los et al. 2009; Noteborn 2009).

In the present study, we took the advantages of both CD22 as a target antigen and apoptin as a cytotoxic moiety to generate a novel antibody conjugate to target CD22 positive leukemia and lymphoma cells. It has been shown that the phosphorylation of specific sites in C-terminus of the apoptin protein is responsible for its nuclear accumulation in cancer cells. In addition there is a strong apoptotic inducing domain in the C-terminus of the protein compared to a weak apoptotic inducing domain in the N-terminal region (Danen-van Oorschot et al. 2003; Kuusisto et al. 2008). Therefore, we prepare an expression cassette in a way to make the target fusion protein with apoptin molecule located at the C-terminus.

The novel recombinant Anti-CD22 scFv–apoptin protein was expressed in *E.coli* BL 21(DE3) using the pET expression vector driven by the strong T7 promoter under optimum expression condition. The protein was successfully purified and refolded with a final yield of 2.7 mg/L and high purity of >90%. Our data also confirmed that the biological activity of scFv fragment was not affected by apoptin and the recombinant fusion protein showed significant interaction with CD22 antigen on the surface of Raji cells. The specific binding to CD22 marker and the subsequent internalization of recombinant anti CD-22–scFv was in accordance with the previous study of CD22 attachment in our laboratory (Zarei et al. 2014).

The efficient delivery of apoptin to the cancer cells has been challenging. It has been shown that apoptin can be introduce to various tumor cells using different delivery strategies in the form of encoding DNA or protein (Los et al. 2009; Peñaloza et al. 2014). Viral-mediated apoptin gene delivery has been widely used to investigate the anticancer activity of apoptin (Pietersen et al. 1999; Pan et al. 2010; Ma et al. 2012; Wu et al. 2011). In addition, bacteria were also used as possible non-viral delivery vehicles of apoptin gene (Guan et al. 2013). In another approach, intra-tumoral administration of plasmid-encoding apoptin via injection or electroporation has also been considered (Lian et al. 2007; Mitrus et al. 2005).

Apoptin could be delivered as an exogenous protein instead of gene-based techniques. Several studies have used fusion proteins including apoptin coupled to a cell membrane-penetrating peptide, like protein transduction domain (PTD) of HIV-1 trans-activator protein (TAT) (Guelen et al. 2004). Likewise, the protein transduction domain 4 (PTD4) demonstrated a similar penetrating effect and has been used in combination with apoptin (Jin et al. 2011; Sun et al. 2009).

To act as a potent apoptosis inducer, apoptin intracellular concentrations must be reached to a threshold level (Peñaloza et al. 2014). Sun et al. reported that after intravenous administration of PTD4–apoptin, the protein uptake by normal cells may limit the efficient accumulation of apoptin in cancer cells (Sun et al. 2009). Hence, the targeted delivery of apoptin would be a solution to overcome this problem. For instance, Peng et al. (Peng et al. 2007) have reported an efficient delivery method of apoptin, based on receptor-mediated endocytosis. Based on their study, the apoptin-encoding DNA was combined with asialoglycoprotein (Asor) to make a protein-DNA complex. They demonstrated that systemic administration of Asor–apoptin resulted in a significant regression on in situ liver tumors in nude mice without apparent cytotoxic effects on the surrounding normal hepatocytes and other normal tissues.

The results of Annexin V/PI flow cytometry analysis in our study clearly revealed that the percentage of apoptotic cells in early and late stages was significantly higher in the CD22 positive Raji cells during 24, 48 and 72 h of incubation with the recombinant protein compared to the CD22 negative Jurkat cells, indicating the involvement of apoptotic machinery activated by apoptin and selective killing of Raji cells and, the efficiency of our targeted delivery approach. Furthermore, while a previous report indicated that tumoral Jurkat cells are very sensitive to apoptin-mediated killing (Maddika et al. 2005), observed resistance of Jurkat cells to the apoptin in our study could be due to the lack of CD22 antigen on the surface of these cells. Consequently, delivery of apoptin and induction of apoptosis was impaired in Jurkat cells as negative control cells.

Our findings highlight the usefulness of anti-CD22 scFv–apoptin protein in specific killing of the target cells. The application of such fusion proteins may overcome some disadvantages of antibody drug conjugates such as unwanted cytotoxicity or detachment of their conjugated drugs due to instability of linkers (Litvak-Greenfeld and Benhar 2012). Moreover, the caspase-dependent apoptosis events induced by apoptin are P53-independent and occur via the intrinsic mitochondrial pathway (Los et al. 2009; Zhuang et al. 1995). It has also been shown that the anti-apoptotic genes products, such as Bcl-2 or Bcr-Abl, do not block the apoptosis induced by apoptin, and even it can be accelerated by overexpression of Bcl-2 (Burek et al. 2006; Los et al. 2009; Noteborn 2004). Conversely, most treatment regimens with chemotherapy agents and radiation rely on the P53 pathway to exert their anti-cancer effects. Considering the P53 gene impairment in many tumor cells, sub-optimal responses to conventional chemotherapy occur (Danen-Van Oorschot et al. 1997; Heilman et al. 2006; Natesan et al. 2006) and may highlight the potential advantage of apoptin as an anti-cancer agent.

In conclusion, the present study has introduced a novel fusion protein for specific targeting of CD22-positive tumor cells. This would be a first step toward a new antibody-based drug against B-cell malignancies.

## Abbreviations

mAb: monoclonal antibody
scFv: single-chain variable fragment
CAV: chicken anemia virus

## References

[CR1] Ahmad ZA, Yeap SK, Ali AM, Ho WY, Alitheen NBM, Hamid M (2012). scFv antibody: principles and clinical application. Clin Dev Immunol.

[CR2] Alderson RF, Kreitman RJ, Chen T, Yeung P, Herbst R, Fox JA, Pastan I (2009). CAT-8015: a second-generation *Pseudomonas* exotoxin A–based immunotherapy targeting CD22-expressing hematologic malignancies. Clin Cancer Res.

[CR3] Becker N, Benhar I (2012). Antibody-based immunotoxins for the treatment of cancer. Antibodies.

[CR4] Bullenkamp J, Tavassoli M (2014). Signalling of apoptin anticancer genes.

[CR5] Bullenkamp J, Gäken J, Festy F, Chong EZ, Ng T, Tavassoli M (2015). Apoptin interacts with and regulates the activity of protein kinase C beta in cancer cells. Apoptosis.

[CR6] Burek M, Maddika S, Burek C, Daniel P, Schulze-Osthoff K, Los M (2006). Apoptin-induced cell death is modulated by Bcl-2 family members and is Apaf-1 dependent. Oncogene.

[CR7] Carnahan J, Wang P, Kendall R, Chen C, Hu S, Boone T, Juan T, Talvenheimo J, Montestruque S, Sun J (2003). Epratuzumab, a humanized monoclonal antibody targeting CD22. Clin Cancer Res.

[CR8] Cesano A, Gayko U (2003). CD22 as a target of passive immunotherapy. Semin Oncol.

[CR9] Coiffier B, Lepage E, Brière J, Herbrecht R, Tilly H, Bouabdallah R, Morel P, Van Den Neste E, Salles G, Gaulard P (2002). CHOP chemotherapy plus rituximab compared with CHOP alone in elderly patients with diffuse large-B-cell lymphoma. N Engl J Med.

[CR10] Danen-Van Oorschot A, Fischer D, Grimbergen JE, Klein B, Zhuang S-M, Falkenburg J, Backendorf C, Quax P, Van der Eb A, Noteborn M (1997). Apoptin induces apoptosis in human transformed and malignant cells but not in normal cells. ‎Proc Natl Acad Sci.

[CR11] Danen-van Oorschot AA, Zhang Y-H, Leliveld SR, Rohn JL, Seelen MC, Bolk MW, van Zon A, Erkeland SJ, Abrahams J-P, Mumberg D (2003). Importance of nuclear localization of apoptin for tumor-specific induction of apoptosis. J Biol Chem.

[CR12] Diamantis N, Banerji U (2016). Antibody-drug conjugates—an emerging class of cancer treatment. Br J Cancer.

[CR13] DiJoseph JF, Armellino DC, Boghaert ER, Khandke K, Dougher MM, Sridharan L, Kunz A, Hamann PR, Gorovits B, Udata C (2004). Antibody-targeted chemotherapy with CMC-544: a CD22-targeted immunoconjugate of calicheamicin for the treatment of B-lymphoid malignancies. Blood.

[CR14] DiJoseph JF, Popplewell A, Tickle S, Ladyman H, Lawson A, Kunz A, Khandke K, Armellino DC, Boghaert ER, Hamann PR (2005). Antibody-targeted chemotherapy of B-cell lymphoma using calicheamicin conjugated to murine or humanized antibody against CD22. Cancer Immunol Immunother.

[CR15] Firer MA, Gellerman G (2012). Targeted drug delivery for cancer therapy: the other side of antibodies. J Hematol Oncol.

[CR16] FitzGerald DJ, Wayne AS, Kreitman RJ, Pastan I (2011). Treatment of hematologic malignancies with immunotoxins and antibody-drug conjugates. Cancer Res.

[CR17] Fulda S, Oster W, Berthold F (1997). Effects of WR-2721 (amifostine) and its metabolite WR-1065 on the antiproliferative activity of chemotherapeutic agents on neuroblastoma cells in vitro. Anticancer Drugs.

[CR18] Furman RR, Coleman M, Leonard JP (2004). Epratuzumab in non-hodgkin’lymphomas. Curr Treat Options Oncol.

[CR19] Grimm S, Noteborn M (2010). Anticancer genes: inducers of tumour-specific cell death signalling. Trends Mol Med.

[CR20] Guan G, Zhao M, Liu L, Jin C, Sun K, Zhang D, Yu D, Cao H, Lu Y, Wen L (2013). *Salmonella typhimurium* mediated delivery of apoptin in human laryngeal cancer. Int J Med Sci.

[CR21] Guelen L, Paterson H, Gäken J, Meyers M, Farzaneh F, Tavassoli M (2004). TAT-apoptin is efficiently delivered and induces apoptosis in cancer cells. Oncogene.

[CR22] Heilman DW, Teodoro JG, Green MR (2006). Apoptin nucleocytoplasmic shuttling is required for cell type-specific localization, apoptosis, and recruitment of the anaphase-promoting complex/cyclosome to PML bodies. J Virol.

[CR23] Hennessy BT, Hanrahan EO, Daly PA (2004). Non-Hodgkin lymphoma: an update. Lancet Oncol.

[CR24] Jin J-l, Gong J, Yin T-j, Lu Y-j, Xia J-j, Xie Y-y, Di Y, He L, Guo J-l, Sun J (2011). PTD4-apoptin protein and dacarbazine show a synergistic antitumor effect on B16-F1 melanoma in vitro and in vivo. Eur J Pharmacol.

[CR25] Keppler-Hafkemeyer A, Kreitman RJ, Pastan I (2000). Apoptosis induced by immunotoxins used in the treatment of hematologic malignancies. Int J Cancer.

[CR26] Krauss J, Arndt MA, Vu BK, Newton DL, Rybak SM (2005). Targeting malignant B-cell lymphoma with a humanized anti-CD22 scFv-angiogenin immunoenzyme. Br J Haematol.

[CR27] Krauss J, Exner E, Mavratzas A, Seeber S, Arndt MA (2009). High-level production of a humanized immunoRNase fusion protein from stably transfected myeloma cells. Methods Mol Biol.

[CR28] Kreitman RJ, Pastan I (2011). Antibody fusion proteins: anti-CD22 recombinant immunotoxin moxetumomab pasudotox. Clin Cancer Res.

[CR29] Kreitman RJ, Pastan I (2015). Immunoconjugates in the management of hairy cell leukemia. Best Pract Res Clin Haematol.

[CR30] Kuusisto HV, Wagstaff KM, Alvisi G, Jans DA (2008). The C-terminus of apoptin represents a unique tumor cell-enhanced nuclear targeting module. Int J Cancer.

[CR31] Leonard JP, Coleman M, Ketas JC, Chadburn A, Furman R, Schuster MW, Feldman EJ, Ashe M, Schuster SJ, Wegener WA (2004). Epratuzumab, a humanized anti-CD22 antibody, in aggressive non-Hodgkin’s lymphoma. Clin Cancer Res.

[CR32] Lian H, Jin N, Li X, Mi Z, Zhang J, Sun L, Li X, Zheng H, Li P (2007). Induction of an effective anti-tumor immune response and tumor regression by combined administration of IL-18 and apoptin. Cancer Immunol Immunother.

[CR33] Litvak-Greenfeld D, Benhar I (2012). Risks and untoward toxicities of antibody-based immunoconjugates. Adv Drug Deliv Rev.

[CR34] Los M, Panigrahi S, Rashedi I, Mandal S, Stetefeld J, Essmann F, Schulze-Osthoff K (2009). Apoptin, a tumor-selective killer. BBA-Mol Cell Res.

[CR35] Ma J-L, Han S-X, Zhao J, Zhang D, Wang L, Li Y-D, Zhu Q (2012). Systemic delivery of lentivirus-mediated secretable TAT-apoptin eradicates hepatocellular carcinoma xenografts in nude mice. Int J Oncol.

[CR36] Maddika S, Booy EP, Johar D, Gibson SB, Ghavami S, Los M (2005). Cancer-specific toxicity of apoptin is independent of death receptors but involves the loss of mitochondrial membrane potential and the release of mitochondrial cell-death mediators by a Nur77-dependent pathway. J Cell Sci.

[CR37] Maddika S, Mendoza FJ, Hauff K, Zamzow CR, Paranjothy T, Los M (2006). Cancer-selective therapy of the future: apoptin and its mechanism of action. Cancer Biol Ther.

[CR39] Malpiedi LP, Diaz CA, Nerli BB, Pessoa A (2013). Single-chain antibody fragments: purification methodologies. Process Biochem.

[CR40] Messmann RA, Vitetta ES, Headlee D, Senderowicz AM, Figg WD, Schindler J, Michiel DF, Creekmore S, Steinberg SM, Kohler D (2000). A phase I study of combination therapy with immunotoxins IgG-HD37-deglycosylated ricin A chain (dgA) and IgG-RFB4-dgA (Combotox) in patients with refractory CD19 (+), CD22 (+) B cell lymphoma. Clin Cancer Res.

[CR41] Mitrus I, Missol-Kolka E, PLUCIENNICZAK A, SZALA S (2005). Tumour therapy with genes encoding apoptin and E4orf4. Anticancer Res.

[CR42] Monnier PP, Vigouroux RJ, Tassew NG (2013). In vivo applications of single chain Fv (variable domain)(scFv) fragments. Antibodies.

[CR43] Natesan S, Kataria J, Dhama K, Bhardwaj N, Sylvester A (2006). Anti-neoplastic effect of chicken anemia virus VP3 protein (apoptin) in Rous sarcoma virus-induced tumours in chicken. J Gen Virol.

[CR44] Nelson AL (2010). Antibody fragments: hope and hype. MAbs.

[CR45] Nitschke L (2005). The role of CD22 and other inhibitory co-receptors in B-cell activation. Curr Opin Immunol.

[CR46] Noteborn MH (2004). Chicken anemia virus induced apoptosis: underlying molecular mechanisms. Vet Microbiol.

[CR47] Noteborn MH (2009). Proteins selectively killing tumor cells. Eur J Pharmacol.

[CR48] Pan Y, Fang L, Fan H, Luo R, Zhao Q, Chen H, Xiao S (2010). Antitumor effects of a recombinant pseudotype baculovirus expressing apoptin in vitro and in vivo. Int J Cancer.

[CR50] Peñaloza OMR, Lewandowska M, Stetefeld J, Ossysek K, Madej M, Bereta J, Sobczak M, Shojaei S, Ghavami S, Łos MJ (2014). Apoptins: selective anticancer agents. Trends Mol Med.

[CR51] Peng D, Sun J, Wang Y, Tian J, Zhang Y, Noteborn M, Qu S (2007). Inhibition of hepatocarcinoma by systemic delivery of apoptin gene via the hepatic asialoglycoprotein receptor. Cancer Gene Ther.

[CR52] Pietersen A, Van der Eb M, Rademaker H, Van den Wollenberg D, Rabelink M, Kuppen P, Van Dierendonck J, Van Ormondt H, Masman D, Van de Velde C (1999). Specific tumor-cell killing with adenovirus vectors containing the apoptin gene. Gene Ther.

[CR53] Potala S, Sahoo SK, Verma RS (2008). Targeted therapy of cancer using diphtheria toxin-derived immunotoxins. Drug Discov Today.

[CR54] Pui C-H, Evans WE (2006). Treatment of acute lymphoblastic leukemia. N Engl J Med.

[CR55] Raut LS, Chakrabarti PP (2014). Management of relapsed-refractory diffuse large B cell lymphoma. South Asian J Cancer.

[CR56] Sant M, Allemani C, Tereanu C, De Angelis R, Capocaccia R, Visser O, Marcos-Gragera R, Maynadié M, Simonetti A, Lutz J-M (2010). Incidence of hematological malignancies in Europe by morphological subtype: results of the HAEMACARE project. Blood.

[CR57] Sullivan-Chang L, O’Donnell RT, Tuscano JM (2013). Targeting CD22 in B-cell malignancies: current status and clinical outlook. BioDrugs.

[CR58] Sun J, Yan Y, Wang XT, Liu XW, Peng DJ, Wang M, Tian J, Zong YQ, Zhang YH, Noteborn MH (2009). PTD4-apoptin protein therapy inhibits tumor growth in vivo. Int J Cancer.

[CR59] Tedder TF, Poe JC, Haas KM (2005). CD22: a multifunctional receptor that regulates B lymphocyte survival and signal transduction. Adv Immunol.

[CR60] Teo EC-Y, Chew Y, Phipps C (2016). A review of monoclonal antibody therapies in lymphoma. Crit Rev Oncol Hematol.

[CR61] Tu X, LaVallee T, Lechleider R (2011). CD22 as a target for cancer therapy. J Exp Ther Oncol.

[CR62] Vallera DA, Brechbiel MW, Burns LJ, Panoskaltsis-Mortari A, Dusenbery KE, Clohisy DR, Vitetta ES (2005). Radioimmunotherapy of CD22-expressing Daudi tumors in nude mice with a 90Y-labeled anti-CD22 monoclonal antibody. Clin Cancer Res.

[CR63] Vallera DA, Todhunter DA, Kuroki DW, Shu Y, Sicheneder A, Chen H (2005). A bispecific recombinant immunotoxin, DT2219, targeting human CD19 and CD22 receptors in a mouse xenograft model of B-cell leukemia/lymphoma. Clin Cancer Res.

[CR64] Wayne AS, FitzGerald DJ, Kreitman RJ, Pastan I (2014). Immunotoxins for leukemia. Blood.

[CR65] Weber T, Mavratzas A, Kiesgen S, Haase S, Bötticher B, Exner E, Mier W, Grosse-Hovest L, Jäger D, Arndt MA (2015). A humanized anti-CD22-onconase antibody-drug conjugate mediates highly potent destruction of targeted tumor cells. J Immunol Res.

[CR66] Weisser NE, Hall JC (2009). Applications of single-chain variable fragment antibodies in therapeutics and diagnostics. Biotechnol Adv.

[CR67] Wu Y, Zhang X, Wang X, Wang L, Hu S, Liu X, Meng S (2011). Apoptin enhances the oncolytic properties of newcastle disease virus. Intervirology.

[CR68] Zarei N, Vaziri B, Shokrgozar MA, Mahdian R, Fazel R, Khalaj V (2014). High efficient expression of a functional humanized single-chain variable fragment (scFv) antibody against CD22 in Pichia pastoris. Appl Microbiol Biotechnol.

[CR69] Zhuang S-M, Shvarts A, van Ormondt H, Jochemsen AG, van der Eb AJ, Noteborn MH (1995). Apoptin, a protein derived from chicken anemia virus, induces p53-independent apoptosis in human osteosarcoma cells. Cancer Res.

